# Predictors of amounts of child and adolescent mental health service use

**DOI:** 10.1007/s00787-022-02063-x

**Published:** 2022-09-16

**Authors:** Julian Edbrooke-Childs, Anisatu Rashid, Benjamin Ritchie, Jessica Deighton

**Affiliations:** 1 Evidence Based Practice Unit, UCL and Anna Freud Centre, 4-8 Rodney Street, London, N1 9JH UK; 2 Child Outcomes Research Consortium, UCL and Anna Freud Centre, London, UK

**Keywords:** Child and adolescent, Mental health, Service use, Case-mix

## Abstract

The aim of this study was to build evidence about how to tailor services to meet the individual needs of young people by identifying predictors of amounts of child and adolescent mental health service use. We conducted a secondary analysis of a large administrative dataset from services in England was conducted using the Mental Health Services Data Set (years 2016–17 and 2017–18). The final sample included *N* = 27,362 episodes of care (periods of service use consisting of at least two attended care contacts and less than 180 days between care contacts) from 39 services. There were 50–10,855 episodes per service. The descriptive statistics for episodes of care were: *M*_age_ = 13 years, SD_age_ = 4.71, range = 0–25 years; 13,785 or 50% male. Overall, there were high levels of heterogeneity in number of care contacts within episodes of care: *M* = 11.12, SD = 28.28, range = 2–1529. Certain characteristics predicted differential patterns of service use. For example, young people with substance use (beta = 6.29, 95% CI = 5.06–7.53) or eating disorders (beta = 4.30, 95% CI = 3.29–5.30) were particularly more likely to have higher levels of service use. To build on this, evidence is needed about predictors of child and adolescent mental health treatment outcome and whether the same characteristics predict levels of improvement as well as levels of service use.

**Supplementary Information:**

The online version contains supplementary material available at 10.1007/s00787-022-02063-x.

## Introduction

One in six young people in England experience mental health difficulties [1]. The focus of the present research is publicly funded child and adolescent mental health services, which are free at the point of contact. THRIVE is the leading model for child and adolescent mental health services in England [2]. Here, service provision is organised into five needs-based groupings: (1) Thriving—those not in need of specialist mental health support; (2) Getting Advice—those in need of advice, signposting to other forms of support, and consultation; (3) Getting Help—those in need of specific goal-focussed support (e.g., a time limited intervention); (4) Getting More Help—those in need of extended and more specialist support; and (5) Getting Risk Support—those with ongoing needs who have not benefited from treatment.

Policy in England calls for child and adolescent mental health services to be tailored to meet the individual needs of young people [3]. One approach to tailoring services is through case-mix classification systems or clustering tools, which identify groups of service users with similar characteristics and therefore, similar levels of service use need [4]. Systematic reviews have identified a range of case-mix characteristics and classification systems for amounts of adult mental health service use [5, 6]. There is less extensive evidence for child and adolescent mental health. A recent systematic review found higher levels of service use in young adults for example, with higher levels of prior service use or those who were female, gay, or bisexual [7]. There is a need for recent evidence from large-scale datasets from child and adolescent mental health services as the most recent data were collected up to 2014 and included up to 11 services [8, 9]. These studies identified high levels of heterogeneity in amounts of service use.

Martin et al. [8] used a conceptual classification system, based on the aforementioned THRIVE model, to identify 18 needs-based groups. Young people in certain sub-groups within the Getting Help and Getting More Help groupings, such as those with depression, self-harm, eating disorder, or psychosis, had higher levels of service use. In addition, young people with comorbid needs (co-occurring behavioural and emotional difficulties or co-occurring emotional difficulties) also had higher levels of service use. However, the relationship between comorbidity and levels of service use is complicated due to the impact comorbidity has on treatment engagement.

Quantitative studies are generally inconclusive about the role of levels of need on premature treatment termination, such as whether higher levels of need are associated with higher levels of premature treatment termination [10–13]. Theory and evidence from qualitative studies indicate a range of reasons for premature treatment termination [14–16]. For example, young people with complex and multiple needs may experience additional challenges to engaging with treatment and may be more likely to have premature treatment termination. Young people may be dissatisfied with treatment, or they may have gained what they needed before reaching the end of treatment, and may be more likely to have premature treatment termination [17].

The aim of the present study was to build on the extant literature and identify predictors of amounts of service use in a large administrative dataset from child and adolescent mental health services in England.

## Methods

### Data preparation and procedure

Data were sourced from routinely collected administrative data, extracted from the ‘community activity data package’ of the Mental Health Services Data Set by NHS Digital (years 2016–17 and 2017–18) [18]. For more information on the dataset including data coverage, see NHS Digital [19]. This package comprises administrative data collected from all publicly funded community services (i.e., not inpatient services) in England. However, only services that submitted data were included, meaning not all services may be represented. No information about services which did not provide data (including how many services did not provide data) are available. A service refers to a collection of mental health teams that provide care over a specified geographical area. From this extract, services that submitted data on care contacts during the 2016–17 and 2017–18 financial years were included if they had at least 50 closed episodes of care.

Care contacts were filtered to include those where the mode of communication was face-to-face, telephone, telemedicine or not stated, and to exclude those where the mode of communication was text message or email; in line with previous research [8], telephone contacts were included to capture direct contact with service users. Episodes of care were constructed, referring to periods of service use consisting of at least two attended care contacts and less than 180 days between care contacts based on session date. We adapted our operationalisation of episodes of care from [20]; here, three care contacts were required, however, we used two because this is in line with national analyses of the present dataset [21]. Episodes of care were included in the present analysis if the age at episode start was 0–25 years, the episode of care was closed, and there was complete data on presenting difficulties (see Measures).

The filtering process is shown in supplementary Fig. 1. There were 50,242,747 care contacts relating to 5,350,642 referrals. We excluded 35,749,942 care contacts (not aged 0–27 years), resulting in 14,492,805 care contacts relating to 1,666,832 referrals. From this, we constructed 459,514 episodes of care (2 care contacts, < 180 days apart, excluded text, email, unattended), meaning 3,683,810 referrals were excluded. Then 34,574 were excluded because young people were aged 26–27 years. This resulted in 424,940 episodes of care, of which 397,578 episodes of care were excluded, mainly because presenting difficulties information was missing. Although there are various definitions of young people and age limits for youth mental health services in the United Kingdom, we restricted the sample in the third step to exclude episodes of care 26–27 years. On reflection, we decided an upper age limit of 25 years was more in keeping with general classification approaches used by services.

This resulted in a final dataset of *N* = 27,362 episodes from 39 services with 50–10,855 episodes per service (*M*_age_ = 13 years, SD_age_ = 4.71, range = 0–25 years; 13,785 or 50% male); please see Table 1 for descriptive statistics on all study variables and supplementary Fig. 1 for full data processing and inclusion flow chart. These episodes of care were for 27,033 young people, meaning < 3% of young people had more than one episode of care during the time period under study. The study was approved by the University College London Research Ethics Committee (12689/001) and the NHS Digital Data Access Request Service (DARS-NIC-140981-R5N6Z). A range of data are collected as part of the ‘community activity data package’ and we included data in accordance with the a priori research questions.

**Table 1:** Descriptive statistics for key study variables

	*n*	%
Demographic characteristics		
Neighbourhood SES		
1 (least)	3908	14.28
2	3835	14.02
3	4430	16.19
4	5672	20.73
5 (most)	9517	34.78
Gender		
Female	13,577	49.62
Male	13,785	50.38
Ethnicity		
Asian	*713*	*2.61*
Bangladeshi	75	0.27
Chinese	55	0.20
Indian	129	0.47
"Other" Asian background	206	0.75
Pakistani	248	0.91
Black	620	2.27
African	263	0.96
Caribbean	185	0.68
"Other" Black background	172	0.63
Mixed-race	*891*	*3.26*
Asian and White	181	0.66
Black African and White	112	0.41
Black Caribbean and White	291	1.06
"Other" mixed-race background	307	1.12
White British	*19,083*	*69.74*
"Other" White (broad, including White Irish)	*722*	*2.82*
White Irish	53	0.19
"Other" White (specific, excluding White Irish)	719	2.63
"Other" ethnic background	848	3.10
Not reported or known	4435.00	16.21
Referral source		
Primary care	10,383	37.95
Other source	4115	15.04
Emergency department	3148	11.51
Education	2126	7.77
Mental health	1895	6.93
Self-referral	1819	6.65
Not reported	1584	5.79
Social care/youth justice	1179	4.31
Child health	1113	4.07
Presenting difficulties		
Internalizing	20,034	73.22
Relational	17,764	64.92
Externalising	12,641	46.20
Neurodevelopmental	11,592	42.37
Self-harm	7465	27.28
PTSD	4877	17.82
Risk management	4601	16.82
Emerging personality disorder	4164	15.22
Eating disorder	3560	13.01
Schizoaffective	3443	12.58
Self-care	2952	10.79
Substance use	2628	9.60

*N* = 27,362 episodes of care for 27,033 young people, from 39 services with 50–10,855 episodes per service. Neighbourhood SES (socio-economic status) was derived using quintiles of the income deprivation affecting children index. Frequencies of the broad ethnic groups (i.e., the sum of the relevant specific groups), used in the main analysis, are highlighted in italics

*PTSD* post traumatic stress disorder

### Measures

#### Deprivation

Deprivation was reported using quintiles of the Income Deprivation Affecting Children Index (IDACI) based on young people’s local area of residence. Local area of residence was available at the level of the Lower Layer Super Output Area, which is the smallest geographic area in England at which deprivation is routinely measured.

#### Demographic characteristics

Age, sex, and ethnicity were recorded by services as part of routine data recording. We refer to sex as our understanding is this variable refers to sex-assigned at birth rather than gender.

Ethnicity was generally reported by the child, young person, or parent/carer at the initial stages of contact with the service and was captured using the categories from the 2001 Census. These were grouped for analysis as follows: Asian (Bangladeshi, Chinese, Indian, Pakistani, “other” Asian background), Black (African, Caribbean, “other” Black background), Mixed-race (White and Asian, White and Black African, White and Black Caribbean, “other” Mixed-race background), White British, “other” White background (White Irish, “other” White background), “other” ethnic background, and not reported or known. We present the broad and specific groups in the descriptive statistics (Table 1), and we used the broad groups in the main analysis to avoid including underpowered groups.

#### Referral route

The individual or organisation making the referral, or the referral source, was recorded by services using 44 categories that were grouped into nine study variables for the present analysis: primary care, self-referral, education, social care/ youth justice, child health, emergency department, mental health, other, and not reported.

#### Presenting difficulties

Two sources were used to identify the presence or absence of 30 non-mutually exclusive presenting difficulties. First, the 30-item clinician-reported Current View questionnaire [22] on presenting problems were used. Second, clinician-reported ICD-10 free-text diagnoses were used. Each ICD-10 diagnosis in the dataset was looked up and then mapped by hand on to the corresponding item of the 30 Current View presenting problems. This was conducted by one author and then reviewed by co-authors in team meetings. New variables were created by transforming the string ICD-10 codes to a Current View presenting problem. The ICD-10 recoded presenting problems when then added to the Current View presenting problems. Episodes of care were included if there was one source of presenting problems, resulting in three possibilities:

1. If an episode of care only had Current View presenting problems, these data points were retained;
2. If an episode of care only had ICD-10 presenting problems, that had been recoded into corresponding Current View presenting problems, these data points were retained;
3. If an episode of care had both Current View and ICD-10 presenting problems, these data points were merged. ICD-10 presenting problems were added to the Current View ones.

Thus, we created one set of harmonised 30 presenting difficulties. Presenting difficulties using these two approaches are typically completed during the initial stages of treatment as part of the initial assessment sessions.

To avoid including an excessive number of presenting difficulty variables in the main analysis, internalising difficulties were grouped together (e.g., social anxiety, low mood), externalising difficulties were grouped together (i.e., conduct disorder, carers having difficulties managing the young person’s behaviour), neurodevelopmental problems were grouped together (i.e., attention-deficit-hyperactivity-disorder, developmental), schizoaffective difficulties were grouped together (i.e., bipolar disorder, psychotic symptoms), and relational problems were grouped together (e.g., peer relationship difficulties, family relationship difficulties). In addition, to avoid including underpowered groups in the main analysis, presenting difficulties with < 10% frequency were excluded: toilet problem, mutism, gender identity, unexplained physical health problems, and adjustment to physical health problems (the episode of care was retained). This resulted in a final set of 12 presenting difficulty variables (please see Table 1).

### Analytic strategy

To investigate predictors of child and adolescent mental health service use, multilevel regressions were performed in STATA 16 [23]. There were two levels in the multilevel regressions, episode of care as the level one group and service as the level two group. As < 3% of young people had more than one episode of care, the analysis was underpowered to include young person as a group. A null model without predictors was computed with number of care contacts as the criterion variable, and the intraclass correlation coefficient (ICC) was calculated. To examine the associations with individual-level characteristics, level-1 predictor variables were added in Model 1: deprivation with the least deprived quintile coded as the reference category to facilitate interpretation; demographic characteristics of grand-mean centred age and female (with male as the reference category as it was marginally the largest group); ethnicity (with White British as the reference category as it was the largest group); referral source with primary care as the reference category as it was the largest group; and the 12 non-mutually exclusive presenting difficulty variables. As the present manuscript is based on a secondary analysis of anonymised administrative data, we do not have further information than that included in the data. There is not additional regional data and we were unable to match to any other service or regional data as these are anonymised. We examined outliers, multicollinearity, and non-normally distributed residuals. All of the predictor variables were binary or categorical, therefore we inspected groups with very low frequencies. There were some low frequencies (< 5%) in ethnicity and referral sources but these were retained due to theoretical importance. After running the multilevel regressions, we plotted the residuals in a Q-Q plot. This indicated some high skew, indicating that lower and higher values were higher than predicted (see Supplementary material). We examined correlations between binary and continuous predictor variables (age, gender, and the presenting difficulty variables). The majority of correlations were significant but none were above 0.7 (the largest was 0.48) (see Supplementary material).

## Results

Overall, there were high levels of heterogeneity in number of care contacts per episode: *M* = 11.12, SD = 28.28, median = 6, range = 2–1,529. As expected based on previous research [8, 24] number of care contacts was non-normally distributed: 18,878 (68.99%) had 2–9 care contacts, 6,832 (24.97%) had 10–29 care contacts, 958 (3.5%) had 30–49 care contacts, and 694 (2.54%) had 50 + care contacts. In the null model, the ICC indicated that 24% of the variance in number of care contacts was explained at the service-level, which remained relatively unchanged in Model 1 (20%). This suggests that almost a quarter of the difference in number of care contacts was due to in which service the episode of care occurred. Model 1 was a significantly better fit to the data than the null model according to the likelihood ratio test: *χ*^2^(32) = 1052, *p* < 0.001. Results of Model 1 are shown in Table 2.

**Table 2:** Multilevel regression with demographic and clinical characteristics predicting number of care contacts

	Beta	SE	*p*-value	95% CI	
Substance use difficulties	**6.29**	0.63	0.00000	5.06	7.53
Eating disorder difficulties	**4.30**	0.51	0.00000	3.29	5.30
Social care/ youth justice vs. primary care referral	**4.17**	0.86	0.00000	2.49	5.85
Mental health vs. primary care referral	**3.98**	0.71	0.00000	2.59	5.37
Relational difficulties	**− 3.25**	0.43	0.00000	− 4.10	− 2.40
Not reported or known vs. WB ethnicity	**− 2.78**	0.49	0.00000	− 3.74	− 1.83
Self-care difficulties	**2.76**	0.56	0.00000	1.67	3.85
Mixed-race vs. WB ethnicity	**2.63**	0.95	0.00600	0.76	4.49
Child health vs. primary care referral	**2.63**	0.89	0.00300	0.89	4.37
Black vs. WB ethnicity	2.16	1.16	0.06200	− 0.11	4.42
Emerging personality disorder	**2.01**	0.51	0.00000	1.02	3.01
Internalising difficulties	**− 2.00**	0.42	0.00000	− 2.83	− 1.18
Schizoaffective difficulties	**1.94**	0.54	0.00000	0.88	3.00
Female vs. male	**1.64**	0.35	0.00000	0.94	2.33
Asian vs. WB ethnicity	1.62	1.06	0.12700	− 0.46	3.71
Education vs. primary care referral	1.19	0.66	0.07400	− 0.11	2.48
Self-referral vs. primary care referral	0.98	0.71	0.16800	− 0.41	2.36
High vs. lowest neighbourhood SES	− 0.95	0.57	0.09800	− 2.07	0.18
Other vs. WB ethnicity	0.73	0.53	0.16600	− 0.30	1.77
Age	**0.71**	0.05	0.00000	0.62	0.80
Self-harm difficulties	− 0.68	0.41	0.10100	− 1.49	0.13
Externalising difficulties	− 0.59	0.41	0.15300	− 1.40	0.22
Emergency department vs. primary care referral	− 0.54	0.57	0.34500	− 1.67	0.58
Not reported or known vs. primary care referral	0.48	0.87	0.58100	− 1.23	2.19
"Other" White background vs. WB ethnicity	− 0.36	1.03	0.72400	− 2.38	1.65
Most vs. least deprived neighbourhood SES	0.19	0.53	0.71700	− 0.85	1.24
"Other" ethnic background vs. WB ethnicity	0.17	1.04	0.86800	− 1.86	2.21
Risk to others difficulties	0.17	0.50	0.73200	− 0.81	1.16
Neuro-developmental difficulties	− 0.07	0.38	0.85500	− 0.82	0.68
Lower vs. least deprived neighbourhood SES	− 0.12	0.62	0.84200	− 1.34	1.09
PTSD difficulties	0.04	0.45	0.92300	− 0.85	0.93
Low vs. least deprived neighbourhood SES	0.03	0.60	0.96600	− 1.15	1.20

*N* = 27,362 episodes of care for 27,033 young people, from 39 services with 50–10,855 episodes per service. Neighbourhood SES (socio-economic status) was derived using quintiles of the income deprivation affecting children index. Coefficients in bold are significant at least at the p < 0.05 level

Results presented in descending absolute beta value

*SE* standard error, *CI* confidence interval

There were no significant effects of economic disadvantage. Regarding demographics, females had higher numbers of care contacts than males, and older young people had lower numbers of care contacts than younger young people. Compared to White British young people, Mixed-race young people had higher numbers of care contacts, and young people with not reported or known ethnic identities had lower numbers of care contacts. Regarding referral source, young people referred through social care/youth justice, child health, or mental health had higher numbers of care contacts than young people referred through primary care.

Regarding presenting difficulties, young people with substance use, eating disorders, self-care difficulties (e.g., personal hygiene), or schizoaffective difficulties had higher numbers of care contacts than young people without these presenting difficulties. Young people with substance use (beta = 6.29) or eating disorders (beta = 4.30) were particularly more likely to have higher levels of service use. Broadly speaking, this suggests for example that episodes of care with substance use as a presenting difficulty had 6.29 more care contacts than episodes of care without substance use, while controlling for other predictors. In contrast, young people with internalising difficulties (beta = − 2.00) or relational difficulties (beta = − 3.25) had lower numbers of care contacts than young people without these presenting difficulties.

## Discussion

The aim of the present study was to build on the extant literature and identify predictors of amounts of service use in a large administrative dataset from child and adolescent mental health services in England. In line with previous studies [8, 9], there were high levels of heterogeneity in amounts of service use, with an average of 11.12 care contacts (SD = 28.28, range = 2–1529). We also found that almost a quarter of the difference in number of care contacts was due to in which service the episode of care occurred.

Regarding presenting difficulties, young people with substance use, eating disorders, self-care difficulties, or schizoaffective difficulties had higher numbers of care contacts than young people without these presenting difficulties, and there were larger effects for substance use and eating disorders. These findings are in line with previous similar research, that found for example associations between psychosis and eating disorders with child mental health service use, although not substance use [8].

In addition, young people with internalising difficulties or relational difficulties had lower numbers of care contacts than young people without these presenting difficulties. Young people with internalising difficulties or relational difficulties may need more specific goal-focussed support or advice, signposting, and consultation such as for peer relationship work in schools or family relationship work in community and voluntary sector services. Alternatively, young people with these difficulties may have been more likely to prematurely terminate treatment, due to experiencing additional challenges to engaging with care, dissatisfaction with care, or having their needs met before the end of treatment [14]. Future research should examine the potential role of comorbidity in number of care contacts.

The findings of the present research are in line with the THRIVE model of child and adolescent mental health service provision in England [2]. Young people with substance use, eating disorders, self-care difficulties, or schizoaffective difficulties may need extended and more specialist support. Future research is needed to examine whether young people with these difficulties are benefitting from longer treatment or whether they have ongoing needs that are not benefited from treatment. Considering that previous quantitative studies have generally been inconclusive about the role of levels of need on premature treatment termination [10–12], future research should examine the association between severity of presenting difficulties, in addition to type of presenting difficulties, and service use. One approach could be to examine the needs-based definition of premature treatment termination, where episodes of care are classified based on whether or not they completed the optimal number of sessions based on levels of need on intake [13, 25].

The findings of the present research add to the literature as they provide recent evidence on child mental health service use from a large administrative dataset. The extant literature suggests that whilst young people with more complex difficulties might require longer treatment durations, they may also be more likely to experience premature treatment termination (see Introduction). The findings of the present research help to address this gap, showing that young people with more complex difficulties had higher numbers of episodes of care. The large amount of variation explained at the service-level (24%) may potentially be explained by the complexity of difficulties, as it may be that services supporting young people with these difficulties were more specialist. For example, substance use had the highest number of episodes of care but was only present for < 10% of young people, and support for substance use may have been provided by dedicated services. When supporting young people with substance use, eating disorders, self-care difficulties, or schizoaffective difficulties, the findings of the present research may suggest that discussing the likelihood that support may take some time, and developing a plan for supporting the young person to remain engaged with support over this time, may help to tailor support to the individual needs of young people.

Young people referred through social care/youth justice had higher numbers of care contacts than young people referred through primary care. Referral through this route is less likely to be voluntary, and it may be that engagement with services was higher as this was compulsory [26]. Young people referred through mental health and child health had higher numbers of care contacts than young people referred through primary care. Referral through these routes may indicate, or be perceived as indicating, a higher level need and correspondingly treatment duration. For example, in another paper examining data from England, when referrals came via mental health, they had a shorter wait than cases referred form primary care [27]. Future research could extend this by examining differences in patterns of service use by referral route. With the increase of remote therapy, future research examining differences in service use by appointment medium would be particularly helpful to informing the tailoring and funding of services.

Young women had higher levels of service use than young men, consistent with the troubling pattern in the literature of higher, and increasing, levels of mental health difficulties for young women [1]. Future research is needed to explain the finding that young people from Mixed-race backgrounds had higher levels of service use than young people from White British backgrounds. Young people with not reported ethnicity had lower levels of service use than young people from White British backgrounds, and future research should examine whether not reporting ethnicity is in and of itself an indicator of challenges with engagement, for example because of a process issue in the service meaning they are not able to collect or record this information or because there is a lack of trust for young people in services meaning they are less likely to report this information.

### Limitations

The limitations of using administrative data should be considered when interpreting the findings of the present research [28]. For example, there may have been differences in how sites collected and coded the data (e.g., referral sources) and services differed in size generally according to the size of the population for whom they cared. Correspondingly, some services may have had more than one team. In addition, the same young person may have been included in different services as young person identifiers were unique within, but not necessarily across, services. Moreover, relatively large amounts of data were excluded at both the service and episode of care levels due to missingness. Although not unusual for administrative data, it does highlight the need to find ways to obtain high quality data on larger percentages of episodes of care receiving public services. There was a lack of data both at the service level and at the regional level, meaning we were unable to examine the influence of these contextual factors on service use. As for other countries, there is a lack of national data on the number of child mental health services, meaning we cannot fully understand how many services are not included in our data or even describe the child mental health system.

Presenting difficulties were identified using two different types of clinician reports, Current View questionnaire [22] presenting problems and ICD-10 free-text diagnoses mapped on to the Current View presenting problems. Although this resulted in a more comprehensive assessment of presenting difficulties than relying on one data source, it may have also resulted in inconsistencies in mapping across sources and moreover, ICD-10 diagnoses may have been inaccurately recorded. In particular, coding was conducted by one researcher, although it was cross-checked by the other researchers in team meetings. Future research should examine predictors of different types of care contacts and professionals involved in care contacts to provide a more granular understanding of the child and adolescent mental health service use needs for young people. This could, for example, include information on the needs-based grouping of young people, based on presenting difficulties [2]. Future research should also go beyond administrative data to follow up young people after they stop using services to examine whether their needs have been sufficiently met or whether they have been unable to continue to use services [14].

The lack of significant association between economic disadvantage and number of care contacts warrants further investigation. It should be noted that a lack of data on structural inequalities may be masking the wider picture on social inequalities [29]. It could be that economic disadvantage has less of an impact on service engagement once support is initiated, whereas young people in areas of higher levels of economic disadvantage may have higher levels of unmet need and be less likely to be referred to mental health services. The indicator of economic disadvantage was based on geographical area, and a more specific indicator of actual family-level economic disadvantage may have shown a different pattern of results.

## Conclusion

Notwithstanding the above limitations, the present research identified predictors of amounts of service use in a large and recent administrative dataset from child and adolescent mental health services in England. Young people with substance use or eating disorders were particularly likely to have higher numbers of care contacts. To build on this, evidence is needed about predictors of child and adolescent mental health treatment outcome and whether the same characteristics predict levels of improvement as well as levels of service use.

### Supplementary Information

Below is the link to the electronic supplementary material.

Supplementary file1 (PNG 90 kb)

Supplementary file2 (PNG 71 kb)

Supplementary file3 (DOCX 22 kb)

## Data Availability

The permission to use the data from NHS Digital prevents sharing of the data.
