# Supplementary figures and images for: Predictors of amounts of child and adolescent mental health service use
Source: Eur Child Adolesc Psychiatry. 2022 Sep 16;32(11):2335–42. doi: 10.1007/s00787-022-02063-x (PMC10576665; doi:10.1007/s00787-022-02063-x)

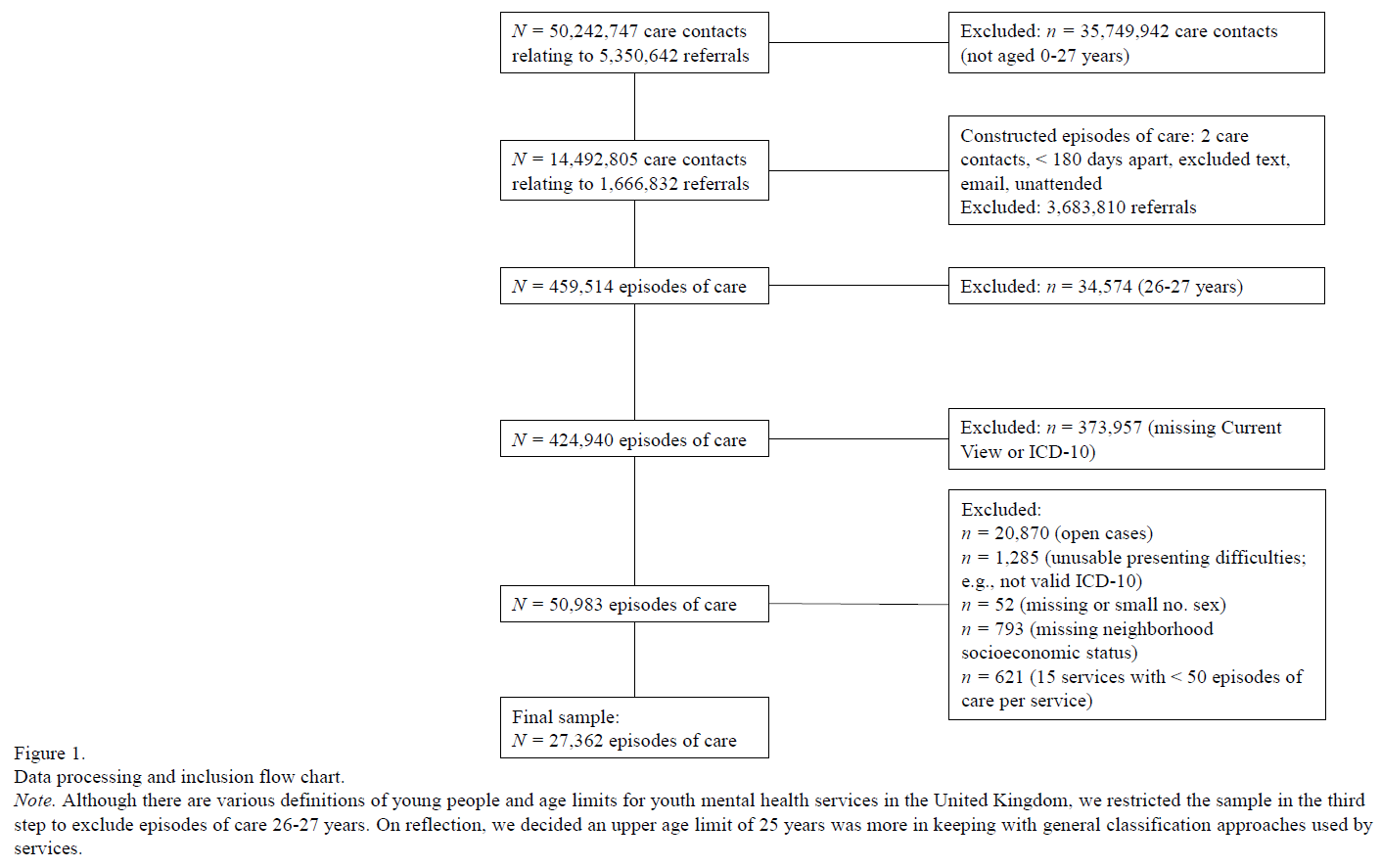

Supplement: Supplementary file 1 — Supplementary file1 (PNG 90 kb) [file 787_2022_2063_MOESM1_ESM.png]

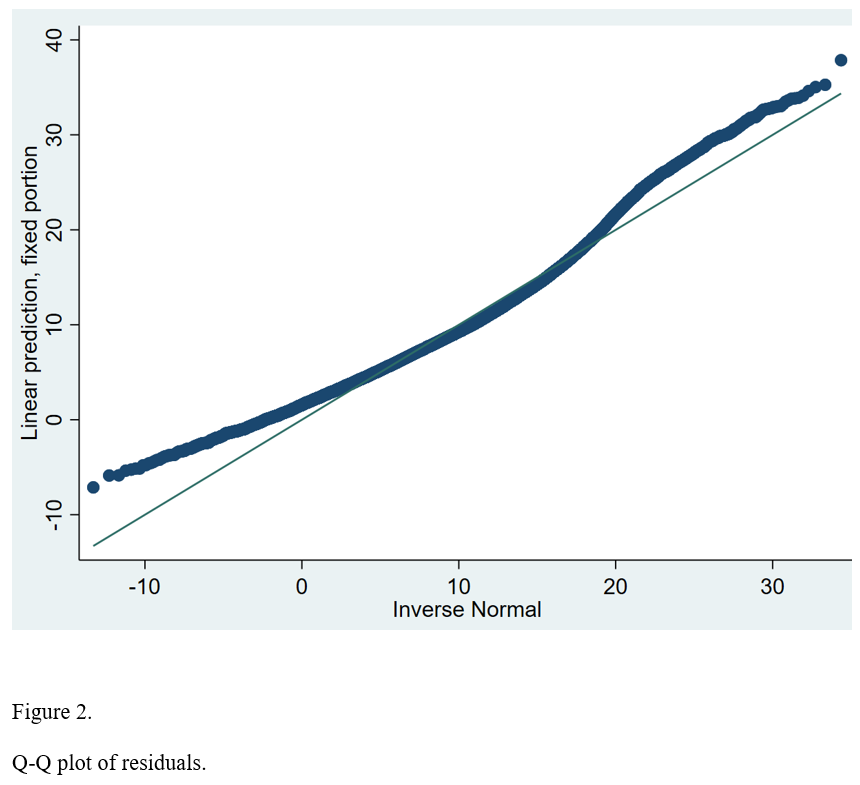

Supplement: Supplementary file 2 — Supplementary file2 (PNG 71 kb) [file 787_2022_2063_MOESM2_ESM.png]
