# Supplementary material for: Predictors of amounts of child and adolescent mental health service use
Source: Eur Child Adolesc Psychiatry. 2022 Sep 16;32(11):2335–42. doi: 10.1007/s00787-022-02063-x (PMC10576665; doi:10.1007/s00787-022-02063-x)
Supplement: Supplementary file 3 — Supplementary file3 (DOCX 22 kb) [file 787_2022_2063_MOESM3_ESM.docx]

**Predictors of amounts of child and adolescent mental health service use**

**Supplementary material**

| Table 1. |  |  |  |  |  |  |  |  |  |  |  |  |  |
| --- | --- | --- | --- | --- | --- | --- | --- | --- | --- | --- | --- | --- | --- |
| Correlation matrix of binary and continuous predictor variables. | | | | | | | | | | | | | |
|  | 1 | 2 | 3 | 4 | 5 | 6 | 7 | 8 | 9 | 10 | 11 | 12 | 13 |
| 1. Gender (female) |  |  |  |  |  |  |  |  |  |  |  |  |  |
| 1. Age | 0.1858* |  |  |  |  |  |  |  |  |  |  |  |  |
| *p*-value | 0.0000 |  |  |  |  |  |  |  |  |  |  |  |  |
| 1. Self-harm | 0.1761* | 0.1056* |  |  |  |  |  |  |  |  |  |  |  |
| *p*-value | 0.0000 | 0.0000 |  |  |  |  |  |  |  |  |  |  |  |
| 1. Substance use | -0.0194* | 0.3579* | 0.0699* |  |  |  |  |  |  |  |  |  |  |
| *p*-value | 0.0014 | 0.0000 | 0.0000 |  |  |  |  |  |  |  |  |  |  |
| 1. Self-care | -0.0167* | -0.0761* | 0.0589* | -0.0254* |  |  |  |  |  |  |  |  |  |
| *p*-value | 0.0058 | 0.0000 | 0.0000 | 0.0000 |  |  |  |  |  |  |  |  |  |
| 1. Risk to others | -0.1626* | -0.1576* | 0.1191* | 0.0362* | 0.1397* |  |  |  |  |  |  |  |  |
| *p*-value | 0.0000 | 0.0000 | 0.0000 | 0.0000 | 0.0000 |  |  |  |  |  |  |  |  |
| 1. Emerging personality disorder | 0.0470* | 0.0680* | 0.1658* | 0.0777* | 0.1278* | 0.2195* |  |  |  |  |  |  |  |
| *p*-value | 0.0000 | 0.0000 | 0.0000 | 0.0000 | 0.0000 | 0.0000 |  |  |  |  |  |  |  |
| 1. PTSD | 0.0808* | 0.0227* | 0.1390* | 0.0300* | 0.0584* | 0.0886* | 0.1382* |  |  |  |  |  |  |
| *p*-value | 0.0000 | 0.0002 | 0.0000 | 0.0000 | 0.0000 | 0.0000 | 0.0000 |  |  |  |  |  |  |
| 1. Eating disorder | 0.1300* | 0.0296* | 0.1482* | -0.0118 | 0.1765* | 0.0329* | 0.1132* | 0.1023* |  |  |  |  |  |
| *p*-value | 0.0000 | 0.0000 | 0.0000 | 0.0516 | 0.0000 | 0.0000 | 0.0000 | 0.0000 |  |  |  |  |  |
| 1. Internalizing | 0.1273* | -0.0042 | 0.2482* | -0.1199* | 0.1050* | 0.0404* | 0.0947* | 0.1665* | 0.1429* |  |  |  |  |
| *p*-value | 0.0000 | 0.4849 | 0.0000 | 0.0000 | 0.0000 | 0.0000 | 0.0000 | 0.0000 | 0.0000 |  |  |  |  |
| 1. Externalising | -0.1405* | -0.3372* | 0.1008* | -0.0428* | 0.1803* | 0.4033* | 0.1723* | 0.1235* | 0.0582* | 0.0963* |  |  |  |
| *p*-value | 0.0000 | 0.0000 | 0.0000 | 0.0000 | 0.0000 | 0.0000 | 0.0000 | 0.0000 | 0.0000 | 0.0000 |  |  |  |
| 1. Neurodevelopmental | -0.2206* | -0.3160* | -0.0275* | -0.0772* | 0.1481* | 0.2456* | 0.1180* | 0.0145* | 0.0437* | 0.0074 | 0.3370* |  |  |
| *p*-value | 0.0000 | 0.0000 | 0.0000 | 0.0000 | 0.0000 | 0.0000 | 0.0000 | 0.0167 | 0.0000 | 0.2186 | 0.0000 |  |  |
| 1. Schizoaffective | 0.0574* | 0.0619* | 0.2181* | 0.0846* | 0.0904* | 0.1771* | 0.2697* | 0.1521* | 0.1448* | 0.1366* | 0.1376* | 0.1065* |  |
| *p*-value | 0.0000 | 0.0000 | 0.0000 | 0.0000 | 0.0000 | 0.0000 | 0.0000 | 0.0000 | 0.0000 | 0.0000 | 0.0000 | 0.0000 |  |
| 1. Relational | 0.0163* | -0.2292* | 0.2503* | -0.0879* | 0.1606* | 0.2463* | 0.1852* | 0.2137* | 0.1395* | 0.3223* | 0.4139* | 0.2175* | 0.1415* |
| *p*-value | 0.0070 | 0.0000 | 0.0000 | 0.0000 | 0.0000 | 0.0000 | 0.0000 | 0.0000 | 0.0000 | 0.0000 | 0.0000 | 0.0000 | 0.0000 |
| *Note. N =* 27,362 episodes of care for 27,033 young people, from 39 services with 50-10,855 episodes per service. PTSD = post traumatic stress disorder. * = significant at least at the *p* < 0.05 level. | | | | | | | | | | | | | |
